# The global burden of climate-sensitive diseases in Brazil: the national and subnational estimates and analysis, 1990–2017

**DOI:** 10.1186/s12963-025-00385-x

**Published:** 2025-07-07

**Authors:** Tatiane C. Moraes de Sousa, Christovam Barcellos, Mauricio L. Barreto

**Affiliations:** 1 Institute of Social Medicine (IMS), State University of Rio de Janeiro (UERJ), Rio de Janeiro, Brazil; 2 Institute of Communication and Information in Health (ICICT), Oswaldo Cruz Foundation (Fiocruz), Rio de Janeiro, Brazil; 3 Institute of Collective Health, Federal University of Bahia (UFBA), Bahia, Brazil; 4 Centre for Data and Knowledge Integration for Health (CIDACS), Oswaldo Cruz Foundation (Fiocruz), Bahia, Brazil

**Keywords:** Climate-sensitive diseases, Climate change, Global Burden of Disease, Vector-borne diseases, Arboviruses

## Abstract

**Background:**

Climatic factors have been associated with the occurrence of several diseases known as climate-sensitive diseases (CSDs). We selected the following categories of disease to represent CSDs for this study: vector-borne diseases (dengue, leishmaniasis, malaria, schistosomiasis, yellow fever, and zika), infectious-diseases (respiratory infections), non-communicable diseases (chronic respiratory and cardiovascular diseases) and water-borne diseases (diarrhea). This study aimed to describe the historical trends and spatial distribution of mortality and morbidity of these selected Climate Sensitive Diseases in Brazil between 1990 and 2017. The analysis is based on findings obtained by the 2017 Brazilian Global Burden of Diseases (GBD) Study.

**Methods:**

Yearly CSD data was taken from the 2017 Brazilian GBD Study for the years between 1990 and 2017. This data was organized by age group and sex at the country level, for 26 states and one Federal District (known as Federative Units), and at the regional level.

**Results:**

Cardiovascular and respiratory diseases presented the greatest disability adjusted life-years (DALYs) in Brazil, followed by chronic and infectious respiratory diseases, although only a small fraction could be attributed to climate changes. Among the vector-borne diseases, the burden of leishmaniasis and malaria have decreased since 1990, while the burden of dengue has increased. The burden of other vector-borne diseases (malaria and yellow fever) increased since 2015, in addition to the recent introduction of zika virus in Brazil. The GBD rates of infectious diseases were greater in predominately the Amazon and northeast regions. This finding contrasts with dengue and zika for which an increase in DALYs rate was observed southeast and central-west, besides the northeast region. The lowest DALYs rates for dengue were observed in the south region, which also experiences the lowest temperatures.

**Conclusions:**

The burden of CSDs in Brazil has increased since 1990 considering non-communicable and communicable diseases. The potential impact of climate change on such diseases must be evaluated considering disease dynamics and spatial specificities, such as land cover and climate patterns. The main challenges in Brazil related to CSDs are the investments needed for research regarding the increase in the burden of CSDs, for vector control and social health determinants mitigation.

**Supplementary Information:**

The online version contains supplementary material available at 10.1186/s12963-025-00385-x.

## Background

Climate-sensitive diseases (CSDs) have been a topic of great interest in health research and for the development of interventions in different regions of the world [1]. The growing interest in CSDs is primarily related to the proven impact of climate change on ecosystems and human health [2]. Climate change’s impacts on health can be direct or indirect [2]. Direct impacts are associated with increased temperature, heat waves, and extreme weather events, with consequences for the incidence of non-communicable diseases, such as cardiovascular and respiratory diseases, communicable diseases, such as diarrhea, and injuries, such as drowning. Impacts from climate change can also occur indirectly, causing environmental degradation, for example, water shortage or contamination, atmospheric pollution, changes in land use, loss of biodiversity, and modification of vector distribution and density [2].

Systemic environmental changes associated with climate change and pre-existing social vulnerability raise concerns about water- and vector-borne diseases. In Brazil, diarrheal diseases have the highest incidence among water-borne diseases. Mortality and morbidity of diarrheal diseases have decreased during the last decades, but their prevalence is still high [3]. In Brazil, dengue, leishmaniasis (visceral and cutaneous), malaria, schistosomiasis, yellow fever, and zika are the most prevalent vector-borne diseases affected by climate change, according to Brazilian health experts [4].

Although the incidence of CSDs is associated with several climate factors [5], pre-existing social, economic, and environmental risk factors also interfere with population health. Several models have been proposed to analyze socioeconomic and climate variables, demonstrating the relation between climate and vulnerable conditions [6, 7].

The Global Burden of Disease (GBD) combines a measure of premature mortality (years of life lost—YLL) with a measure of disability (years lived with disability—YLD) in order to derive an overall measure of disease burden (disability adjusted life-years—DALYs) [8]. The GBD Study presents mortality and morbidity of major diseases, injuries, and health risk factors at global, national, and regional levels. The 2017 Brazilian GBD Study provided values for all GBD indicators (i. e., YLL, YLD, and DALYs) since 1990 for Brazilian subnational administrative levels, which included 26 states and one Federal District (collectively known as Federative Units) by sex and age group.

Climate change has been a risk factor in GBD studies since 2000, indicating the growing concern about increasing greenhouse gas (GHG) emissions on different population health outcomes [9]. Since then, different studies have proposed to measure the impacts of climate variables and climate change on human health using the GBD methodology [10].

This range of factors act as health determinants in different ways according to environmental and social characteristics, demanding a better understanding of the temporal and spatial distribution of CSDs in Brazil. This article aims to analyze the distribution of CSDs in Brazil, considering 27 subnational administrative sectors (26 states and one federal district) and five regions (north, northeast, southeast, south, and central-west) between 1990 and 2017.

Many studies have analyzed the relationship between climatic factors and specific infectious diseases [11]; however, this is the first study to utilize GBD metrics to analyze a set of CSDs in Brazil.

## Methods

### Global Burden of Disease study overview

The 2017 Brazilian GBD Study provided the data used in this paper. The Brazilian GBD research group is coordinated by the Institute for Health Metrics and Evaluation (IHME) at the University of Washington in collaboration with the Brazilian Ministry of Health and the Federal University of Minas Gerais (UFMG).

The main data sources in GBD estimates are vital record systems, vernal autopsy data, cancer data registries, surveillance data for maternal mortality, injuries, and child death, and census and surveys for maternal mortality and injuries [12]. Mortality data was derived from the Mortality Information System (Sistema de Informação sobre Mortalidade–SIM). Morbidity data was derived from Notifiable Disease Information System (Sistema de Informação de Agravos de Notificação–SINAN), Hospital Information System of the Brazilian Unified Health System (Sitema de Informações Hospitalares do Sistema de Único de Saúde-SIH/SUS) and Outpatient Information System of the Unified Health System (Sistema de Informações Ambulatoriais do SUS-SIA/SUS). In addition, there are specific disease databases that were used in our GBD estimate, such as the Schistosomiasis Control Program Information System (Sistema de Informação do Programa de Controle da Esquistossomose–SISPCE) and the Epidemiological Surveillance System for Malaria (Sistema de Vigilância Epidemiológica da Malária – SIVEP Malária).

The Brazilian GBD Study presented data from 1990 to 2017. This data compiled annual estimates of mortality and morbidity for 333 diseases and injuries at national and sub-national levels, comprised of 26 states and the Federal District (known collectively as the Federative Units). In addition, we also present the estimates for all Brazilian macro-regions: north, northeast, southeast, south and central-west. The DALYs are also analyzed by seven age groups (< 1; 1–4; 5–14; 15–49; 50–69; > 70) and sex (male and female). The results are presented in standardized rates (per 100,000 inhabitants) by sex, age group, year, and Federative Units or regions.

When analyzing from a spatial perspective, we note that Brazilian Federative Units and regions have a wide range of socioeconomic conditions, access to healthcare and urban infrastructure, and income and education [13, 14]. This diversity results in different epidemiological profiles.

### Selection criterion of the climate sensitive diseases

An initial list of CSDs was obtained from the National Observatory of Climate and Health (www.climaesaude.icict.fiocruz.br), which carried out three workshops with Brazilian experts to identify the most relevant CSDs for Brazil [4].

The list of CSDs obtained during this workshop was combined with an extensive literature review on CSDs for the world and Brazil [5]. According to this review, the most studied CSDs were cardiovascular, respiratory, and vector-borne diseases, primarily dengue and malaria. In addition to these diseases, we included diarrheal diseases, leishmaniasis, and schistosomiasis because these diseases were considered important CSDs by Brazilian health experts. We also included two other arboviruses, yellow fever, and zika, due to their importance in Brazil starting in 2015.

For the temporal analyses, we aggregated vector-borne diseases (dengue, malaria, leishmaniasis, schistosomiasis, yellow fever, and zika) and considered the 27 Federative Units and five regions; for the spatial analysis, we divided the CSDs between Communicable and Noncommunicable diseases.

In addition to temporal and spatial analysis, the contributions of mortality (YLL) and morbidity (YLD) indicators to DALY estimates of the CSDs were also analyzed, as were the main climate variables of Brazilian regions, namely temperature and precipitation.

### Brazilian climate zones

In Brazil there is a longitudinal gradient of rainfall reduction to central Brazilian areas, with the highest precipitation values in the western Amazon area and in almost all of the eastern coastal areas. As for annual mean temperature values, there is a gradient of reduction in the north–south direction, with the highest temperature values recorded in the north and northeast and some coastal areas in northeast and southeast regions [15].

**Fig. 1:**
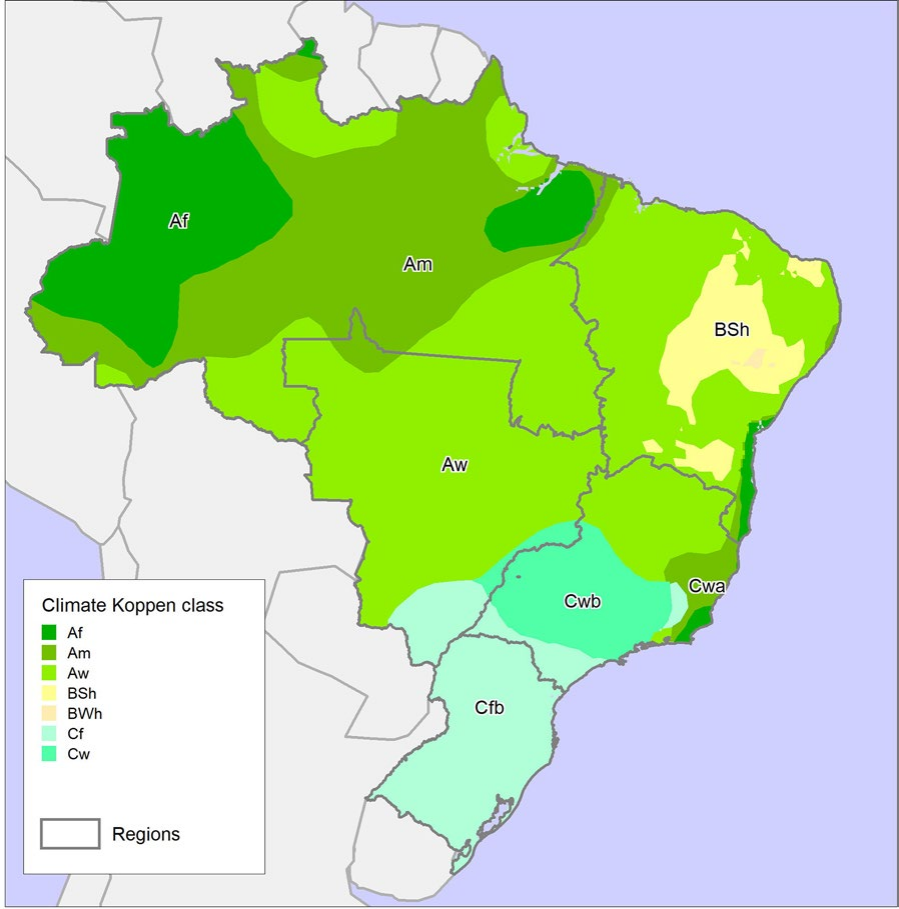
Climate zones according to Köppen criteria location according to Brazilian regions

Considering the climate classification proposed by Köppen (1936) presented in Fig. 1 and Table 1, the main climate zones in Brazil are tropical, humid subtropical and dry [15]. However, the rainfall and temperature patterns are not homogeneous in these climate zones. The Tropical zone has areas without a dry season and others with a dry winter or summer. In the Amazon, there is also a great tropical monsoon zone, according to the Köppen classification. The tropical zone covers all of the north region, almost all of the central-west and parts of the northeast and southeast, mainly in their coastal areas. The humid subtropical zone is predominant in the southeast and south; however, there are several differences in temperature and precipitation values. Dry winters are found in almost all of the southeast region, while the south does not have a dry season [15].

**Table 1:** The main characteristics of the climate zones according to Köppen criteria and the predominantly Brazilian regions of occurrence [12]

Climate zones	Main characteristics	Brazilian regions
Tropical climate zone (Af, Am, Aw)	All months have average temperatures above 18 °C and precipitation > 60 mm and < 25 mm	North, Northeast, Southeast and Central-West
Dry Semiarid Zone (BSh)	Temperature > 18 °C with deficient precipitation (> 5 and < 10 mm) during most of the year	Northeast
Humid Subtropical Zone (Cwa, Cwb, Cfb)	Temperature > − 3 °C and < 22 °C and precipitation > 40 mm during most of the year	South and Southeast

## Results

### CSD trends

**Fig. 2:**
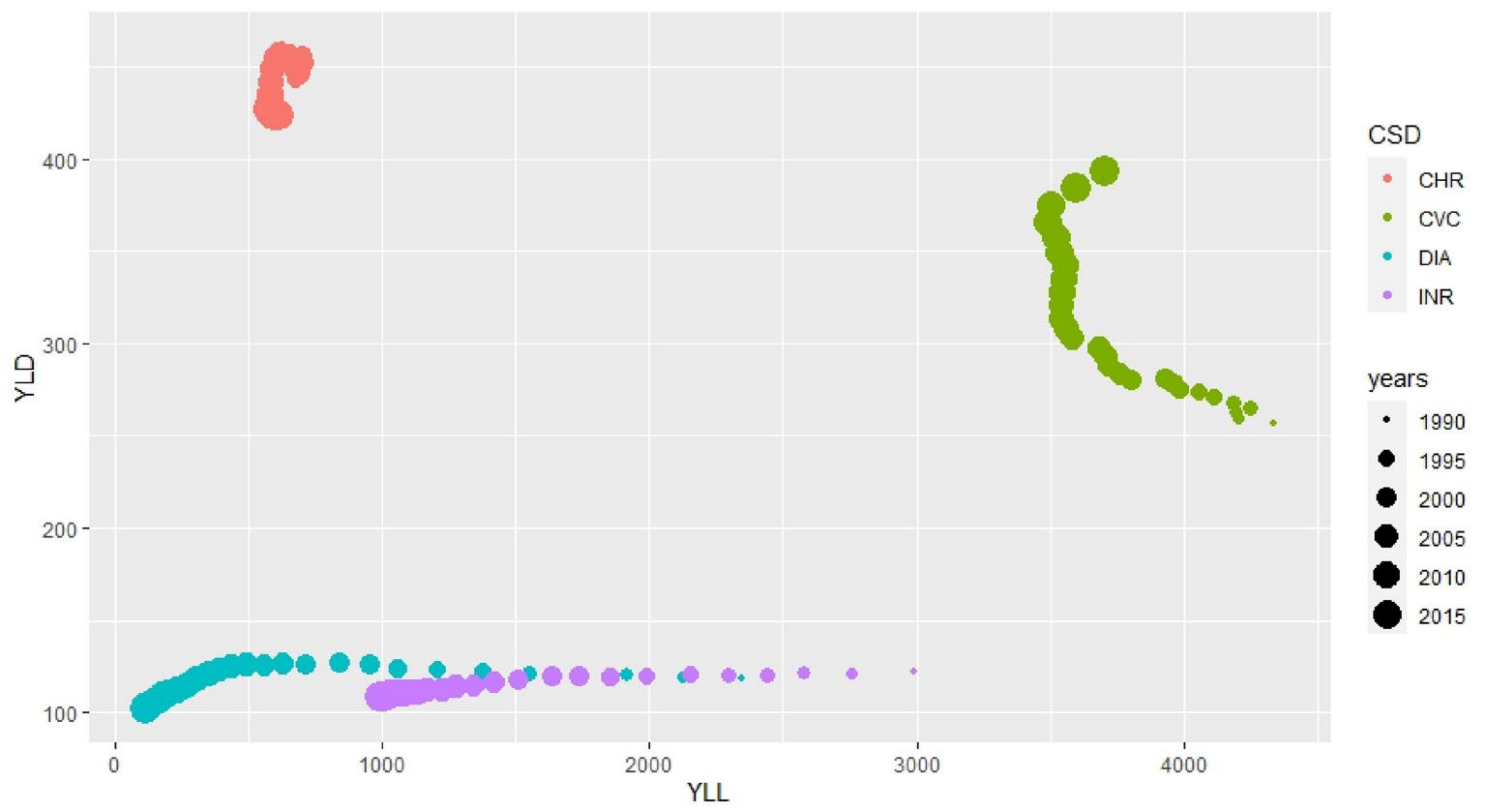
Evolution of the YLL and YLD rates per 100,000 inhabitants age adjusted for CSD with highest values between 1990 and 2017 in Brazil (CHR = Chronic Respiratory Diseases, CVC = Cardiovascular Diseases, DIA = Diarrheal Diseases, INR = Respiratory Infections and Tuberculosis)

**Fig. 3:**
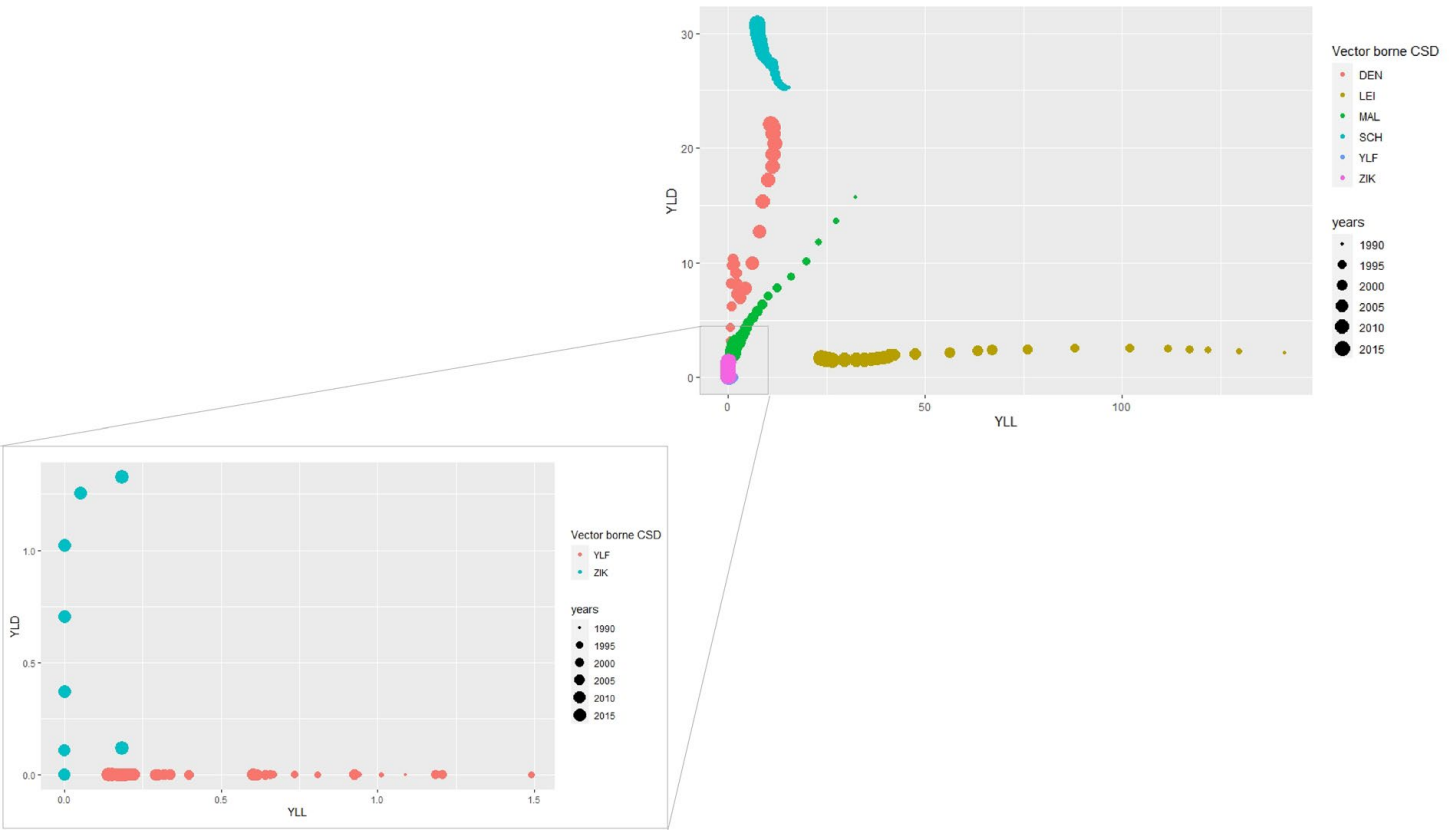
Evolution of the YLL and YLD rates per 100,000 inhabitants age adjusted for vector-borne CSD and, in detail, for yellow fever and zika between 1990 and 2017 in Brazil (DEN = dengue, LEI = leishmaniasis, MAL = malaria, SCH = schistosomiasis, YLF = yellow fever, ZIK = zika)

Table 2 shows the age standardized rates per 100,000 inhabitants of DALY, YLL and YLF for all CSDs for all Brazilian regions in 1990 and 2017, while Figs. 2 and 3 present the evolution of the mortality (YLL) and morbidity (YLD) components of DALYs of all of the selected CSDs in Brazil over the 1990–2017 period. According to Fig. 2, four groups of disease showed higher burdens if we consider both DALY’s components (YLL and YLD): (i) cardiovascular diseases; (ii) chronic respiratory diseases; (iii) infectious respiratory diseases, and (iv) diarrheal diseases. Cardiovascular diseases presented high values in both periods, mainly due to an increase in mortality rates (YLL). Chronic and infectious respiratory diseases ranked second and third among all CSDs. Diarrheal diseases ranked fourth among all CSDs, even though the number of DALYs declined since 1990, mainly due to a decrease in YLL.

**Table 2:** Age adjusted rate per 100,000 inhabitants and uncertainty interval (95% UI) of DALY, YLL and YLD of the Climate Sensitive Diseases in 1990 and 2017 for all Brazilian Regions (CVC = Cardiovascular diseases, CHR = Chronic respiratory diseases, DEN = Dengue, DIA = Diarrheal diseases, LEI = Leishmaniases, INR = Respiratory infections and tuberculosis, SCH = Schistosomiasis, YLF = Yellow Fever, ZIK = Zika virus)

		CVC		CHR			DEN		DIA		LEI		MAL		INR		SCH		YLF		ZIK	
REGIONS		**1990**																				
		N	UI	N	UI	N		UI	N	UI	N	UI	N	UI	N	UI	N	UI	N	UI	N	UI
DALY	North	2480	2084—2874	761.7	632–891	1.16		1.02–1.3	2702	1735–3670	357.4	−250–965	761.5	254,5–1268	2714	1939–3490	6.46	−5.7- 18,6	5.035	0.25–9.82	0	0
	Northeast	3721	3295–4147	951	836–1067	2.07		1.5–2.6	5668	3953–7386	291.3	115–468	8.07	1.01–15.14	5009	3900–6118	25.9	8.05–43.8	1.5	−1.23–4.3	0	0
	Southeast	5462	3259–7666	1148	843–1454	1.51		1.03–1.2	885	720.7–1049	56.13	−63.4–175.7	5.01	3.5–6.5	2306	1216–3395	48.01	2.67–93.53	0.5	0.3–0.67	0	0
	South	4742	3201–6283	1326	863–1789	0.76		0.51–1.02	778	−186.5–1742	2.17	−0.37–4.72	5.7	1.7–9.7	1601	1340–1863	7.35	−6.7–21.4	0.4	0.3–0.5	0	0
	Central-west	3313	2557–4070	857.8	632.2–1083	1.32		1.02–1.62	978.6	391.3–1565	219.5	−142.2–581.2	82	−123–287	1672	1102–2241	65.2	−60.1–190.5	1.33	018–2.48	0	0
YLL	North	2313	1926–2700	384.4	289–480	0.381		0.329–0.434	2559	1627–3491	347	−262–956	502	171–833	2590	1817–3362	2.836	−3.63–9.35	5.035	0.25–9.82	0	0
	Northeast	3493	3068–3919	524.2	432.3–616.1	1.02		0.5–1.5	5498	3792–7205	288	113–464	3.12	−0.55–6.8	4880	3772–5988	14.6	6.3–23	1.5	−1.23–4.34	0	0
	Southeast	5178	3038–7318	674.7	414.6–934.7	0.43		0.27–0.6	797	649.5–945.2	55.2	−63.5–174	1.66	0.85–2.46	2187	1097–3278	18.74	1.81–35.7	0.48	0.29–0.67	0	0
	South	4468	2982.5–5953	817	459.2–1175	0.32		0.3–0.34	681	−254.5–1616	1.5	0.23–2.8	2.5	−0.9–5.9	1484	1227.3–1740	5.12	−4.84–15.1	0.41	0.27–0.55	0	0
	Central-west	3127	2403–3850	446.1	239–653.2	0.36		0.33–0.39	874.6	317.8–1431.4	216.4	−145.7–578.4	75.5	−120.5–271.6	1552	986.1–2118	12.47	−11.6–36.6	1.33	0.18–2.5	0	0
YLD	North	166	150–182	377	341–413	0.78		0.65–0.91	143	105–180	10.2	4.5–16	259	71.8–447	124.6	121.4–127.8	3.6	−2.4–9.6	0.002	−0.0003–0.0036	0	0
	Northeast	229	211–244	427.5	403.2–451.8	1.05		0.9–1.2	171.2	156–186	2.7	1.13–4.28	4.9	1.56–8.3	129	126–131	11.3	0.8–21.7	0.0003	−0.00025–0.001		
	Southeast	284	219–349.3	474.3	421.6–527	1.08		0.75–1.4	87.5	67.2–107.8	0.9	−0.05–1.9	3.4	2.61–4.1	118	117–119	29.35	−3.6–62.3	0.0001	0.00009–0.0002	0	0
	South	275	194.7–354.7	509.3	403.2–615.4	0.44		0.21–0.677	97.03	67.05–127	0.66	−083–2.16	3.17	2.48–3.86	118	112.6–123.2	2.23	−1.95–6.41	0.0001	0.00009–0.0001	0	0
	Central-west	187	151–22.8	411.7	384.4–439.1	095		0.66–1.24	104	68.6–139-4	3.16	−1.5–7.8	6.5	−2.4–15.5	120	114.2–125.1	52.8	−53.7–159.2	0.0004	0.00006–0.0007	0	0
REGIONS		**2000**																				
		N	UI	N	UI	N		UI	N	UI	N	UI	N	UI	N	UI	N	UI	N	UI	N	UI
DALY	North	264	52.06–38.6	76.14	64.8–87.5	3.4		2.7–4.15	28.6	21.7–35.4	6.2	−2.8–15.4	2.05	1.21–2.9	101	83.8–118.1	0.6	−0.46–1.6	0.05	0.007–0.1	0.02	0.006–0.03
	Northeast	391	360–432	88	73.4–102.4	4.2		3.5–5	37	32–42	5.2	1.8–8.5	0.2	0.2–0.3	111.7	105.4–118	2.28	0.67–3.9	0.01	−0.002–0.02	0.03	0.008–0.05
	Southeast	479	369.6–589.2	111	102.7–120.3	4.15		1.8–6.45	15.6	8.3–23	0.98	−1.12–3	0.22	0.13–0.3	121.3	79–163.5	4.4	−0.045–8.9	0.01	0.006–0.02	0.04	−0.04–0.12
	South	412	287.3–536.6	131	86.15–175.7	1.24		0.2–2.28	14.2	7.5–20.8	0.04	−0.04–0.1	0.2	0.17–0.21	85.9–61.9	109.9	0.5	−0.5–1.6	0.001	0.009–0.01	0.001	−0.001–0.003
	Central-west	338	228–448.5	92.5	70.3–115-4	4.4		2.5–6.3	18	8.2–27.7	4.07	−3.3–11.4	0.4	−0.1–0.9	82.2	49.5–115	6.2	−6.23–18.64	0.017	0.015–0.02	0.07	−0.1–0.22
YLL	North	237	191.5–282.3	41.5	31.6–51.5	1.5		1–2.03	17.6	12.8–22.4	5.12	−4.1–14.3	0.98	0.5–1.4	89.4	72.6–106	0.16	−0.21–0.5	0.054	0.007–0.1	0.01	0.004–0.02
	Northeast	361	324.6–397	50.3	37.4–63.2	1.55		1.1–2	23	17.8–28	5	1.7–8.2	0.08	0.03–0.12	99.7	93.4–106	0.88	0.5–1.3	0.01	−0.002–0.02	0.02	0.004–0.03
	Southeast	433	326.8–540	64.7	55.8–73.6	1.34		−0.27–2.41	7.13	4.12–10.1	0.94	−1.12–3	0.06	0.04–0.08	110	66.66–154	0.85	0.14–1.56	0.013	0.006–0.02	0.02	−0.02–0.07
	South	369.4	2582.2–486.6	83	46.7–119.4	019		−0.44–0.84	5.5	1.75–9.35	0.025	0.0012–0.05	0.05	0.047–0.07	75.8	52–99.7	0.28	−0.51–0.51	0.012	0.009–0.013	0.0005	−0.0006–0.001
	Central-west	306	201–411	52.7	30.7–74.7	2.13		0.85–3.42	8.73	2.05–15.4	3.86	−3.6–11.3	0.2	−0.2–0.6	71.7	39.6–103.7	0.5	−0.54–1.56	0.017	0.015–0.20	0.041	−0.054–1.56
YLD	North	27.05	24.2–30	34.6	32.8–36.3	1.9		1.6–2.2	11	6.7–15	1.05	0.4–1.7	1.07	0.6–1.5	11.5	11.1–12	0.41	−0.27–1.1	0.00002	0.000003–0.00003	0.007	0.002–0.01
	Northeast	35.4	33.3–37.3	37.6	35.8–39.5	2.64		2.3–3	14	12–16	02	−0.11–0.5	0.16	0.13–0.2	12	11.5–12.4	1.4	0.05–2.7	0.0000003	−0.0000006–0.0000007	0.01	0.003–0.02
	Southeast	46	41.6–50.3	46.8	39.01–54.6	2.8		1.5–4	8.5	3.7–13.3	0.03	−0.01–0.08	0.15	0.093–0.2	11.12	8.5–13.8	3.6	−0.55–7.7	0.000005	−0.000002–0.0000008	0.02	−0.02–0.05
	South	42.6	33.1–52	47.8	39.1–56.6	1.04		0.5–1.6	8.6	5.7–11.5	0.02	−0.04–0.08	0.14	0.12–0.15	10.10	9.9–10.3	0.3	−0.3–0.8	0.000004	0.000003–0.000004	0.0004	−0.0008–0.002
	Central-west	33.30	27–37.7	40.1	39.4–40.8	2.3		1.66–2.9	9.2	5.3–13	0.2	−0.27–0.69	0.17	0.1–0.24	10.5	9.8–11.3	5.7	−6.12–17.5	0.000005	0.0000027–0.0000079	0.03	−0.05–0.11

Figure 3 depicts the burden of vector-borne diseases much smaller than the top four CSDs (cardiovascular, chronic and infectious respiratory and diarrheal diseases). Among vector-borne diseases, the largest increase in DALYs is found for dengue over the 1990–2017 period, with the largest values in YLL and YLD. Another vector-borne disease whose burden increased in the period was schistosomiasis; however, with higher morbidity contribution (YLD).

The vector-borne diseases with the greatest decrease in DALYs were leishmaniasis (mainly due to mortality reduction) and malaria, for which a substantial decline in mortality and morbidity was reported. Considering all CSDs included in this study, the diseases with the lowest DALYs were yellow fever and zika, both with higher YLL than YLD.

### Spatial CSDs distribution

**Fig. 4:**
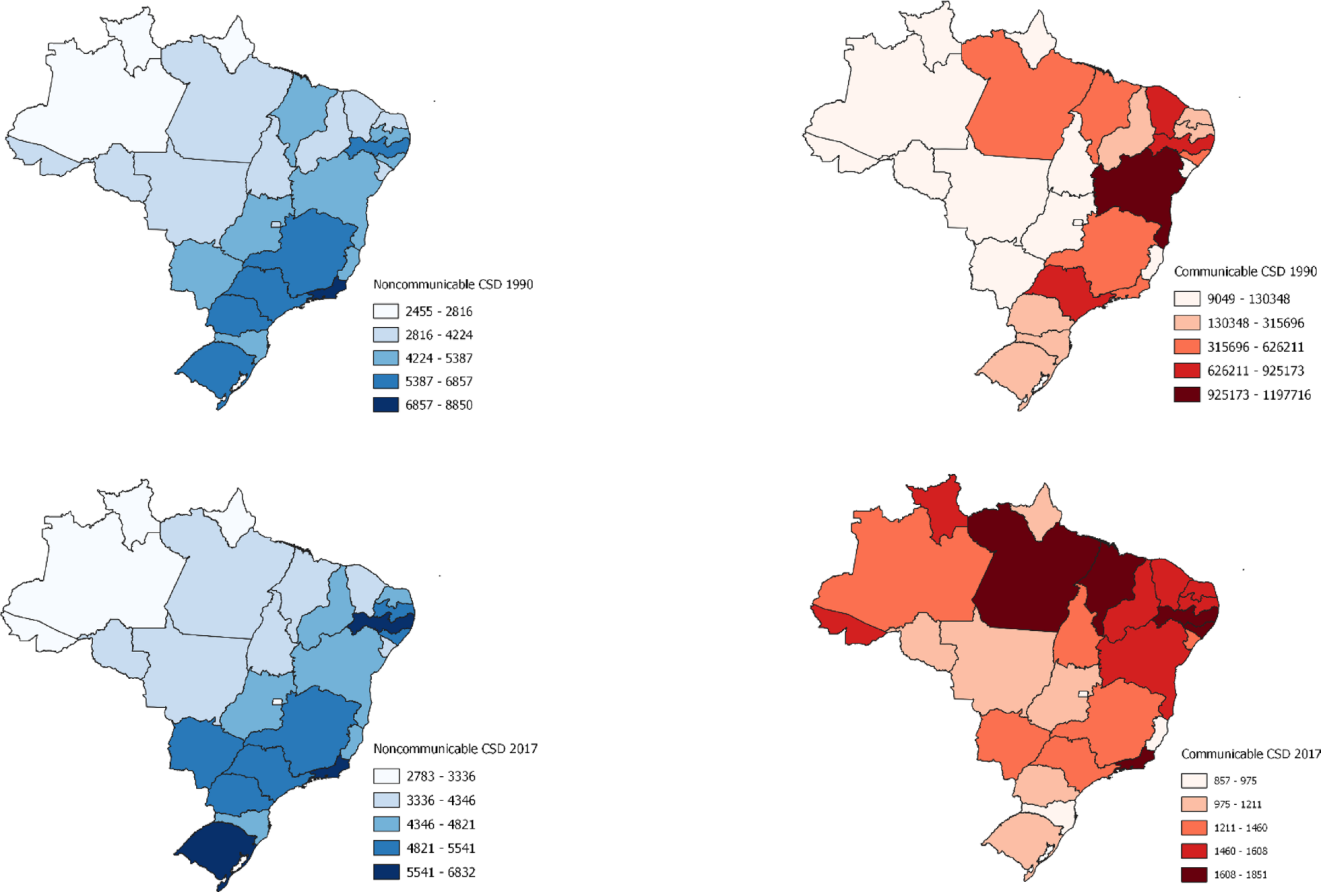
Spatial distribution of the DALY rates (per 100,000 inhabitants) for noncommunicable and communicable CSDs in Brazil in 1990 and 2017 (each map presents a different scale)

Figure 4 presents the DALY rates of communicable and noncommunicable CSDs for the Brazilian Federative Units in 1990 and 2017. Noncommunicable cardiovascular and chronic respiratory diseases (NCDs) had higher DALY rates in 1990 and 2017 in the states located in the southeast, south, and northeast regions.

The DALY rates of communicable diseases (CDs) decreased in the studied period, and they were more distributed among the states of the north region, such as Pará, and the northeast, such as Pernambuco. There is also a relevant global burden of CDs in states in the southeast, the most populous Brazilian region, and the central west, which has a vast rural area.

**Fig. 5:**
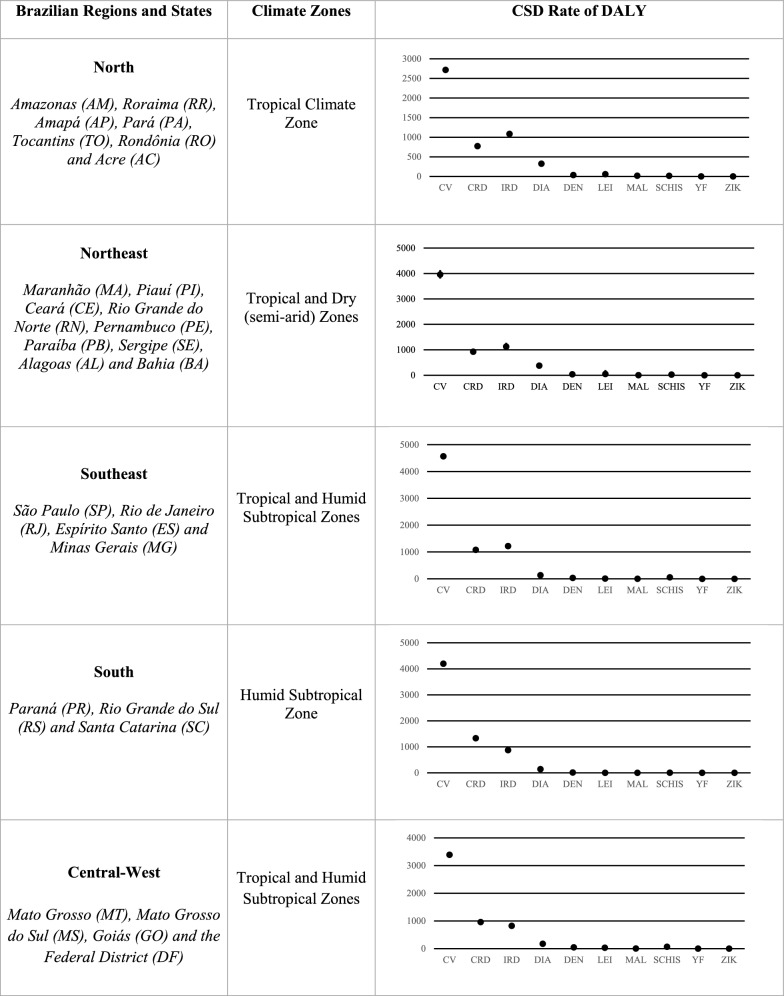
CSD total number of DALYs, per 100,000 hab., for all Brazilian regions in 2017 according to Köppen Climate Zones

Figure 5 illustrates the DALY rates per 100,000 inhabitants of the CSDs for each region in 2017 according to Köppen criteria (Table 1). According to Fig. 5, cardiovascular diseases presented the highest DALYs in 2017 for all Brazilian regions, followed by infectious and chronic respiratory diseases. Chronic respiratory diseases had the largest number of DALYs in the south and central-west, while infectious respiratory diseases presented the highest values in the north, northeast, and south. Diarrhea was the fourth CSD with the highest number of DALYs in all Brazilian regions; however, its highest value was found in the northeast, where the only semi-arid zone in Brazil is located. There were also high numbers of DALYs of diarrhea in the southeast and south regions.

**Fig. 6:**
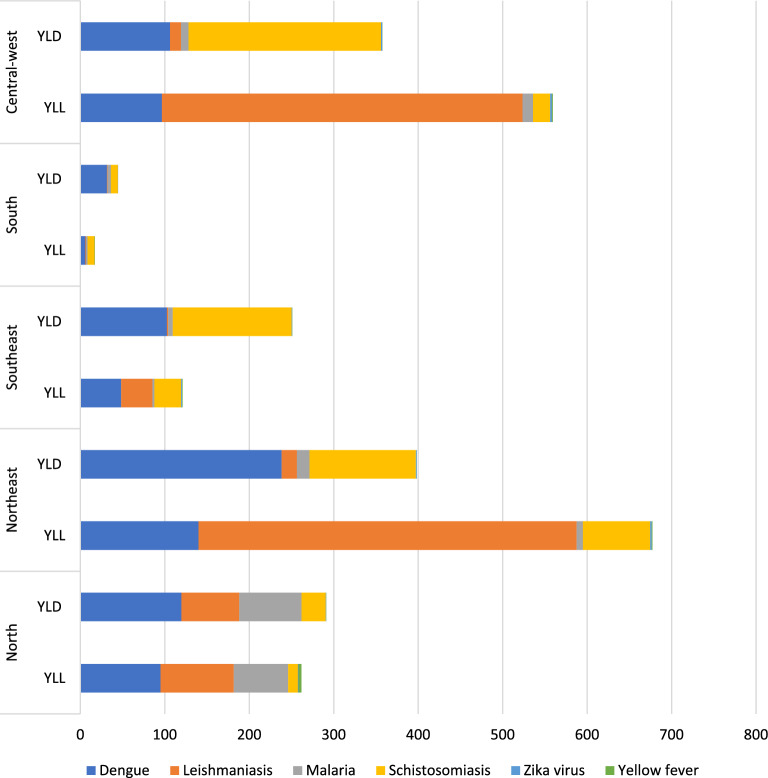
YLL and YLD contribution to DALYs for water- and vector-borne CSDs for Brazilian regions in 2017

To understand the global burden distribution of vector-borne diseases, Fig. 6 shows the composition of the DALYs (i.e., the percentage of YLL and YLD in the GBD composition) of these diseases for each Brazilian macroregion in 2017.

When analyzing the distribution of dengue, the regions with the greatest number of DALYs in 2017 were the northeast, north, and central-west. While dengue presents higher YLD values, mortality is also high (Fig. 6). Although these regions are composed of diverse climate zones [15], their most populated areas record dry winters and high precipitation values during the summer (humid subtropical zone). The climate patterns presented in the area covered by the humid subtropical zone are strongly associated with the occurrence and spread of the dengue vector (*Aedes* spp.) [11]. Schistosomiasis presented the highest values in the central-west, northeast, and southeast, mainly due to morbidity estimates (YLD).

Considering maximum and minimum DALY values estimated in 2017, leishmaniasis presented the highest range, mainly in the northeast and central-west regions. The highest DALYs for malaria were found in the north, with similar values for YLL and YLD (Fig. 5), although malaria was also recorded in other regions (Fig. 5).

Finally, the CSDs with the lowest DALYs in 2017 were yellow fever and zika for all Brazilian regions, although yellow fever presented higher values in the north and central-west, while zika presented higher values in the southeast, northeast, and central-west regions. The global burden study results obtained for yellow fever and zika are discussed and compared with Brazilian Ministry of Health information below, in the Discussion section.

### CSDs global burden in Brazil by sex and age groups

**Fig. 7:**
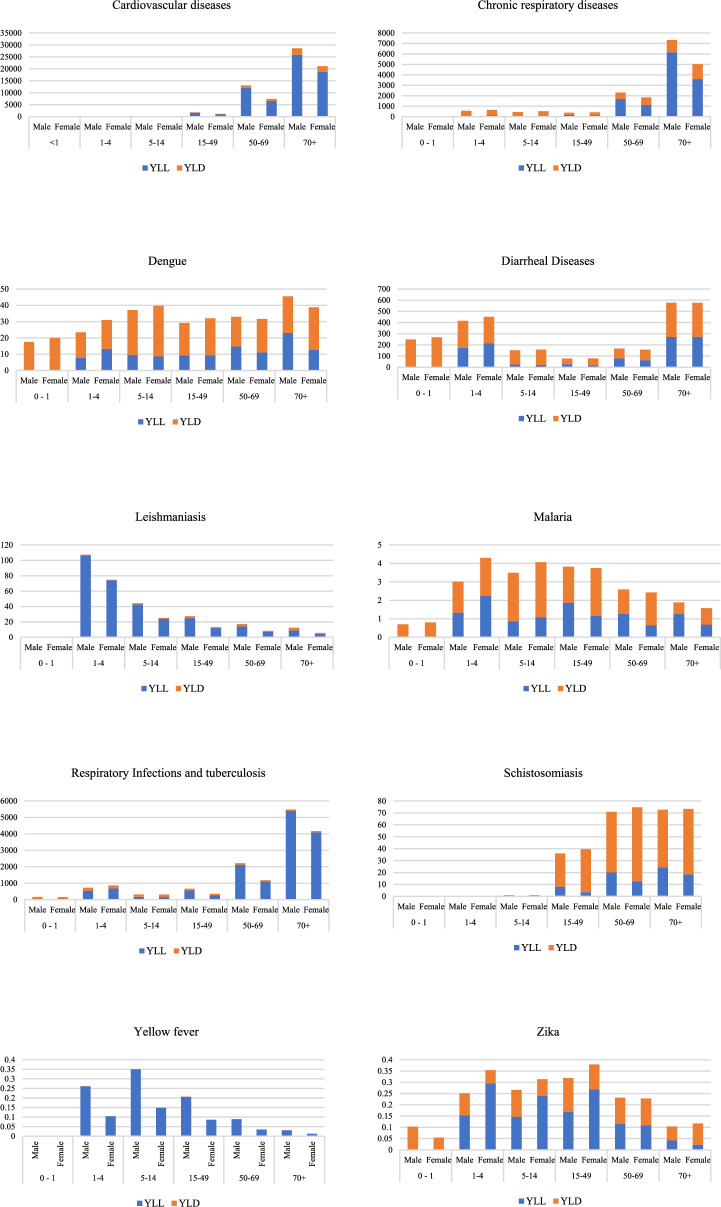
Number of DALYs, YLLs and YLDs for all CSD for Brazil in 2017 according to age groups and sex

Figure 7 presents the standardized rate of YLL and YLD of all CSDs for Brazil in 2017 according to seven age groups (< 1; 1–4; 5–14; 15–49; 50–69; > 70) and sex (male and female). Cardiovascular diseases presented the highest values in adults, mainly those over 50 years old. Chronic and infectious respiratory diseases impacted all age groups; however, the highest-burden values were associated with adults over 70 years old. When the CSD global burden is analyzed by sex, cardiovascular and respiratory diseases show the highest DALY, mostly among males, composed mainly of premature deaths. It is worth noting that chronic respiratory diseases are primarily associated with tobacco smoking, while climate can play a secondary role (or trigger acute episodes).

As for diarrheal diseases, the largest DALYs are seen in children (< 4) and older people (> 70), with global burden of diarrhea values for all age groups. The DALYs of diarrhea presented higher morbidity (YLD) than mortality (YLL), with similar values for both sexes.

DALYs of vector-borne diseases were found for all age groups except for schistosomiasis, which presented a global burden for people over 15 years old. When the DALYs distribution is analyzed by sex, there are evident differences for leishmaniasis and yellow fever, with higher DALYs for adult males and zika with higher values for adult females.

## Discussion

Among the diseases covered in this study, cardiovascular, infectious, and chronic respiratory diseases presented a greater global burden, followed by diarrheal diseases, in the country and all regions. Among the vector-borne diseases, we highlight the high burden of leishmaniasis throughout the study period and the increased burden of schistosomiasis in Brazil and dengue between 1990 and 2017. Concerning the composition of the DALYs, we note a higher weight of mortality (YLL) in the global burden of cardiovascular and infectious respiratory diseases, diarrhea, leishmaniasis, and yellow fever. Morbidity (YLD) was higher in the composition of the global burden of dengue and schistosomiasis, while the YLL and YLD indicators of malaria, zika, and infectious respiratory diseases presented similar weight in the DALYs composition. During the study period, there was a decrease in the mortality component (YLL) of diarrheal diseases, schistosomiasis, leishmaniasis, malaria, and yellow fever without reducing incidence (YLD). On the other hand, there was an increase in both mortality (YLL) and morbidity (YLD) for dengue, chronic respiratory diseases, and cardiovascular diseases.

The results presented illustrate the particular form the epidemiological transition of the Brazilian population has assumed over the last decades [16]. Chronic noncommunicable diseases present a high burden, which is characteristic of developed countries. In contrast, infectious and parasitic diseases, associated with vulnerable socioeconomic conditions, continue to be significant in some regions of the country, as opposed to showing the expected reduction over time. The recent emergence and trends of some climate-related transmissible diseases, in particular arboviruses, are added to this diverse epidemiological profile [17–20].

According to data from the Brazilian GBD Study, the CSDs with the highest number of DALYs, both in absolute numbers and rates, are cardiovascular diseases and infectious diseases (chronic and infectious). Although cardiovascular diseases are associated with different risk factors, such as aging, obesity, and smoking, among others, there is also evidence of a strong association between the increase in the occurrence of these diseases and changes in climatic variables. The main climatic variables associated with these diseases are the increase in ambient temperature, precipitation, and heat and cold waves [21–23].

Chronic and infectious respiratory diseases are also associated with different social and environmental determinants of health. These environmental determinants of health include climatic factors, mainly temperature and humidity [24–27], as well as air pollution from sources such as automotive vehicles in large cities and forest fires in forest and rural areas [28] which can aggravate the occurrence of these diseases.

It should be emphasized that both chronic and infectious respiratory diseases can occur with greater severity in vulnerable populations, such as children and the elderly [24, 29], in addition to more vulnerable social groups, such as those with low income [30].

Although cardiovascular and respiratory diseases are a public health concern due to their high burden in Brazil, diseases transmitted by water and vectors are a priority for the Brazilian health system both because of their persistent burden and the worsening of some outcomes and the emergence of new ones in recent years [16].

The global burden of diarrhea, albeit with a reduction since 1990, still has a high number of DALYs across the country. Although the DALYs of diarrhea are mainly composed of the morbidity indicator (YLD), there is still an expressive value of early mortality (YLL) associated with this outcome. The persistence of diarrhea in Brazil is mainly due to the fragility of coverage of health facilities in the north and northeast [3] and socioeconomic factors, such as low schooling and poor housing conditions [31]. In recent years, outbreaks of diarrhea have been reported in some semi-arid states in the northeast region, mainly due to the occurrence of drought episodes that have aggravated access to quality drinking water [32, 33]. On the other hand, mortality, and therefore the YLL of diarrhea, is strongly influenced by the coverage and quality of primary health care services, which can diagnose and treat this group of diseases promptly [34].

In relation to vector-borne diseases, the incidence of dengue increased during the entire study period, and the burden reduction trend for malaria and yellow fever reversed in the last two or three years. Two new arboviruses, zika and chikungunya, appeared in the country but were absent from GBD studies. It should be noted that among the vector-borne CSDs, those that presented a continuous decrease in cases were schistosomiasis and leishmaniasis.

Schistosomiasis occurs in a large part of Brazil, presenting endemicity on the northeast coast, in addition to the states of Espírito Santo and Minas Gerais in the southeast region [35, 36]. The relationship of the climate with the incidence of schistosomiasis is mainly related to the influence of temperature, precipitation, and humidity on the population density of its host, the snail [37–40], aggravated by a lack of sanitation services.

Leishmaniasis had a greater burden than schistosomiasis, being among the diseases transmitted by vectors and having the highest burden in Brazil, although this has reduced since 1990. In Brazil, cases have been recorded of both types of leishmaniasis, cutaneous leishmaniasis and visceral leishmaniasis, both occurring in rural and urban areas of the country [41]. The greater global burden is in the states of the north, northeast, and central-west regions.

Dengue was the CSD that presented the largest increase in global burden in Brazil in the studied period, mainly due to the introduction of different serotypes of virus (DEV1, DEV2, DENV3, and DENV4) in the country since 1990 [17, 42]. In addition to this introduction of the different serotypes, the area of occurrence of the main vector of the disease in Brazil, the *Aedes aegypti* mosquito, also expanded [11]. Thus, the incidence of dengue is directly related to temperature and precipitation, especially in February, March, and April, at the end of the Brazilian summer, when the highest temperatures and rainfall rates occur [11, 43]. Dengue is a major public health concern due mainly to the high number of cases with a high morbidity burden (YLD) and a high incidence of deaths (YLL) due to the severe forms of the disease [44].

While dengue has been a public health concern since the nineteenth century, when the first epidemic was recorded in Brazil [45], the first case of zika was recorded in Brazil in 2015 and has since become a priority due to its severity, which has become greater than dengue in some aspects [18, 46, 47]. Although it presents similar symptoms to those of other arboviruses, such as fever, rash, and joint pain, zika also has a strong association with congenital malformations and neuropathies such as microcephaly and neurological problems, such as Guillan-Barré Syndrome [48–50].

The number of DALYs associated with zika in Brazil refers mainly to the morbidity of adult individuals, with the highest number of cases recorded in the northeast, southeast, and central-west regions.

Yellow fever was the other arbovirus, with a recent increase in cases in Brazil [51]. From the 1940 s until the middle of 2014, yellow fever was concentrated in the Amazon region, which was considered endemic. However, as of 2014, human cases and epizootics were recorded in other areas, initially in the north, which includes the Amazon biome, and later in the central-west and southeast regions [19]. In 2016 and 2017, there was an outbreak of the disease in Brazil, mainly in the states of São Paulo, Minas Gerais, and Rio de Janeiro [19]. These states have occupation characteristics and urban land cover that differ from the endemic region, although the vectors were the same, and there was sylvatic transmission of the disease [19, 52].

Besides the increase in the spatial extent of the disease, there was also an increase in the number of cases [51]. Between the period considered in this study, 1990 and 2016, less than 100 cases of yellow fever had been recorded per year; in 2017, more than 800 cases were recorded, reaching 1376 cases in 2018 [19]. Among the explanations for the recent outbreak of yellow fever in Brazil has been the lack of universal vaccine coverage and environmental impacts that have altered the natural areas of occupation of non-human primates, which are hosts of the yellow fever virus [51].

Finally, in relation to malaria, Brazil, similar to other Latin American countries, recorded a 59% increase in autochthonous cases in 2017 compared to 2016 [20] after a period of reduced disease transmission. The cases of malaria recorded in Brazil occurred mainly in the Amazon region, with a higher incidence in the states bordering other countries [53]. The persistence of malaria in the Amazon region, as well as the recent increase in cases, is due to a diversity of environmental and socioeconomic determinants, such as the deforestation of some areas and the predominance of economic activities that increase the risk of exposure to vectors (irregular agriculture and mining, as well as unplanned occupation of land) [54].

### Brazilian GBD study assessment

The use of the results obtained by the 2017 Brazilian GBD Study in the CSD evaluation allowed for comparison between these outcomes over time and space considering all the states of the country, given the availability of subnational data, which has shown to be an excellent advance in health measurability.

The availability of data on morbidity (YLD) is also crucial to studying CSDs, especially for water- and vector-borne diseases. These are historically neglected health outcomes. With the advancement of the coverage of health structures and health services, there has tended to be a reduction in mortality but a lower morbidity reduction, which is often overlooked. Thus, using the YLD indicator in the epidemiological evaluation of these diseases allows for identifying priorities that can be masked if mortality is used exclusively as an indicator of population health.

Among the limitations of using the GBD identified in this study is the absence of chikungunya, a disease that has been a high burden in Brazil since 2015 and has recurrent outbreaks in different regions of the country [55]. The disease has low lethality but may present a high YLD load due to the sequelae left by central nervous system inflammation that can last for a few years [56]. The chikungunya virus is also transmitted by the mosquito *Aedes aegypti*, the vector of dengue and zika, making a synergistic analysis of these three diseases necessary to promote interventions and control policies for these arboviruses [57].

The results of the GBD Study presented a global burden of zika since 2013, while the first case registered in Brazil was in 2015 [47]. There were also two differences concerning yellow fever in GBD and national data. While the GBD database indicated a higher burden in the states of the north and south regions, national data suggest a higher number of disease cases recorded in the southeast region [19]. Another difference is the weight of early mortality (YLL) in the disease burden 2017. According to the GBD Study, more than 95% of DALYs related to yellow fever are due to YLL; according to the Brazilian Ministry of Health, there were about 1376 cases reported in 2017, of which 483 died, so about 35% of reported cases resulted in mortality [19].

These divergences demand a constant review of the sources used by the GBD Study.

We found a large distinction between the contribution of each indicator, YLL and YLD, in the DALY composition of each disease, including regional variations in the case of arboviruses. Overall, the lower weight of morbidity (YLD) was verified when it could be higher. The low values of YLD are generally due to deficiencies in reporting these diseases, both with morbidity and mortality, as well as underreporting of diarrheal and arboviruses diseases [58, 59]. In relation to arboviruses, there is still confusion about diseases that present some similar symptoms, such as dengue, zika, and chikungunya [60]. However, although notification problems occur, it is still necessary to better understand the approximations in estimating DALYs concerning some diseases and regions. Refinement of the GBD methodology and its sources of information (in this case, the system of reporting of diseases and mortality information system) would promote improvements in estimating the Global Burden of Disease and national population health data.

### The Brazilian challenges related to CSD

The increasing global burden of CSDs makes this group of diseases a public health priority in Brazil. In addition to being relevant to the health of the Brazilian population, arboviruses are also very important in other countries. The extent of these diseases in Latin America and Asia, especially in border areas, requires analysis and joint actions with other countries and international bodies [61].

Although the occurrence of CSDs is due to various variables, the reduction of the lethality of these outcomes is directly associated with the effective presence of health services and with the availability of adequate assistance. Thus, the retraction in primary health care services coverage may increase the number of deaths and hospitalizations associated with these outcomes [62].

The Brazilian scenario of reducing investments in public systems, including the health system, due to the approval by the National Congress in 2016 of Constitutional Statement No. 95, which determines the freezing of public expenditures for 20 years, may result in aggravation in the control of morbidity and mortality of these diseases [62].

The main limitation of this study is related to the notification of climate-sensitive diseases, mainly the data on morbidity that provides the YLD estimation according to GBD methodology. Another limitation is the association between the global burden of climate-sensitive diseases and climate patterns. In this paper, we used the Köppen climate classification; however, there is no perfect match between the Köppen climate classes of climate and the Brazilian regions. In this way, improving the impact of climate factors and climate change on the burden of climate-sensitive diseases is fundamental.

## Conclusions

According to the results of the Brazilian GBD Study, the global burden of CSDs has increased in Brazil since 1990, mainly concerning cardiovascular diseases, respiratory diseases, and dengue. The emergence of Zika in 2015 and the worsening of malaria and yellow fever cases in 2016 should also be noted. The higher burden also refers to mortality due to the possible underestimation of the morbidity of these outcomes. Cardiovascular and respiratory diseases presented the highest number of DALYs in 2017 in all Brazilian Federative Units. For arboviruses, the spatial distribution varies according to each disease, with dengue and zika having cases recorded in different regions, with a lower incidence in the coldest parts of the country, especially in the south region. On the other hand, malaria is restricted to the Amazon region, and yellow fever, with a predominant transmission in the Amazon region, had the highest number of recent cases in the southeast region, where urban occupation is predominant.

## Supplementary Information

Supplementary material 1.

## Data Availability

The data we used in this article are publicly available on the official websites of the Institute of Health Metrics and Evaluation (http://ghdx.healthdata.org/gbd-results-tool) and the Health Informatics Department of the Brazilian Ministry of Health (DATASUS).

## Abbreviations

GBD: Global Burden of Disease
YLL: Years of life lost
YLD: Years lived with disability
DALY: Disability adjusted life years
CSD: Climate sensitive diseases
FU: Federal units
IHME: Institute of Health Metrics and Evaluation
UFMG: Universidade Federal de Minas Gerais
Fiocruz: Oswaldo Cruz Foundation
INPE: National Institute of Spatial Researches
CVD: Cardiovascular diseases
CRD: Chronic respiratory diseases
IRD: Infection respiratory diseases
DD: Diarrheal diseases
NCD: Noncommunicable diseases
CD: Communicable diseases
COPD: Chronic obstructive pulmonary disease
DEV: Dengue virus
