# Supplementary material for: The global burden of climate-sensitive diseases in Brazil: the national and subnational estimates and analysis, 1990–2017
Source: Popul Health Metr. 2025 Jul 7;23(Suppl 1):29. doi: 10.1186/s12963-025-00385-x (PMC12231730; doi:10.1186/s12963-025-00385-x)
Supplement: Supplementary file 1 — Supplementary material 1. [file 12963_2025_385_MOESM1_ESM.docx]

Supplementary Material

Age adjusted rate per 100,000 inhabitants of DALY, YLL and YLD of the Climate Sensitive Diseases in 1990 and 2017 for all Brazilian Federal Units (FU) and Brazil (CVC = Cardiovascular diseases, CHR = Chronic respiratory diseases, DEN = Dengue, DIA = Diarrheal diseases, LEI = Leishmaniases, INR = Respiratory infections and tuberculosis, SCH = Schistosomiasis, YLF = Yellow Fever, ZIK = Zika virus)

| **DALY (Disability Adjusted of Lost Years)** | | | | | | | | | | | | | | | | | | | | | | | | | | | | | | | | | | |
| --- | --- | --- | --- | --- | --- | --- | --- | --- | --- | --- | --- | --- | --- | --- | --- | --- | --- | --- | --- | --- | --- | --- | --- | --- | --- | --- | --- | --- | --- | --- | --- | --- | --- | --- |
|  | 1990 | | | | | | | | | | | | | | | | | 2017 | | | | | | | | | | | | | | | | |
|  | **CVC** | **CHR** | **DEN** | **DIA** | | **LEI** | | **MAL** | | **INR** | | **SCH** | | **YLF** | | **ZIK** | | **CVC** | | **CHR** | | **DEN** | | **DIA** | | **LEI** | | **MAL** | | **INR** | | **SCH** | **YLF** | **ZIK** |
| Acre | 2633 | 996 | 1 | | 3666 | | 24 | | 1304 | | 4010 | | 0 | | 4 | | 0 | | 2413 | | 923 | | 28 | | 346 | | 23 | | 30 | | 1134 | 0 | 0 | 0 |
| Alagoas | 4218 | 1178 | 2 | | 10333 | | 83 | | 5 | | 7513 | | 42 | | 0 | | 0 | | 4333 | | 902 | | 47 | | 500 | | 16 | | 2 | | 1252 | 33 | 0 | 1 |
| Amapá | 1885 | 578 | 1 | | 1113 | | 12 | | 524 | | 1485 | | 0 | | 3 | | 0 | | 2170 | | 629 | | 47 | | 188 | | 14 | | 25 | | 936 | 0 | 0 | 0 |
| Amazonas | 2096 | 727 | 1 | | 3099 | | 15 | | 306 | | 2434 | | 0 | | 4 | | 0 | | 2119 | | 664 | | 28 | | 322 | | 6 | | 15 | | 1035 | 0 | 0 | 0 |
| Bahia | 3522 | 979 | 1 | | 5646 | | 190 | | 5 | | 4239 | | 28 | | 1 | | 0 | | 3856 | | 966 | | 28 | | 412 | | 33 | | 2 | | 1110 | 22 | 0 | 1 |
| Ceara | 3223 | 891 | 2 | | 6556 | | 496 | | 5 | | 5916 | | 6 | | 0 | | 0 | | 3407 | | 788 | | 47 | | 318 | | 81 | | 2 | | 1112 | 7 | 0 | 0 |
| Distrito Federal | 3022 | 738 | 1 | | 584 | | 142 | | 10 | | 1282 | | 159 | | 1 | | 0 | | 2589 | | 728 | | 28 | | 104 | | 21 | | 2 | | 540 | 162 | 0 | 0 |
| Espírito Santo | 4329 | 943 | 2 | | 1018 | | 13 | | 6 | | 1557 | | 24 | | 1 | | 0 | | 4989 | | 1154 | | 60 | | 204 | | 3 | | 3 | | 1015 | 20 | 0 | 0 |
| Goiás | 3502 | 1057 | 2 | | 854 | | 46 | | 31 | | 1567 | | 102 | | 2 | | 0 | | 3475 | | 1055 | | 56 | | 158 | | 6 | | 3 | | 825 | 86 | 0 | 0 |
| Maranhão | 3952 | 912 | 4 | | 5833 | | 521 | | 32 | | 6297 | | 24 | | 11 | | 0 | | 3649 | | 697 | | 43 | | 425 | | 92 | | 5 | | 1155 | 17 | 0 | 0 |
| Mato Grosso | 2840 | 779 | 1 | | 1464 | | 136 | | 275 | | 2142 | | 0 | | 1 | | 0 | | 3207 | | 941 | | 47 | | 213 | | 26 | | 8 | | 902 | 0 | 0 | 2 |
| Mato Grosso do Sul | 3891 | 856 | 1 | | 1013 | | 554 | | 12 | | 1697 | | 0 | | 1 | | 0 | | 4260 | | 991 | | 46 | | 242 | | 109 | | 2 | | 1023 | 0 | 0 | 0 |
| Minas Gerais | 4791 | 1166 | 1 | | 911 | | 166 | | 5 | | 1932 | | 76 | | 1 | | 0 | | 4063 | | 1062 | | 33 | | 188 | | 29 | | 2 | | 1041 | 68 | 0 | 0 |
| Pará | 2664 | 791 | 1 | | 4241 | | 354 | | 473 | | 3361 | | 35 | | 3 | | 0 | | 2893 | | 804 | | 35 | | 374 | | 59 | | 14 | | 1230 | 30 | 0 | 0 |
| Paraíba | 4101 | 1026 | 2 | | 2624 | | 76 | | 4 | | 3886 | | 11 | | 0 | | 0 | | 4524 | | 920 | | 37 | | 326 | | 14 | | 2 | | 1140 | 13 | 0 | 0 |
| Paraná | 4813 | 1195 | 1 | | 1155 | | 3 | | 7 | | 1707 | | 10 | | 0 | | 0 | | 4157 | | 1249 | | 17 | | 171 | | 1 | | 2 | | 861 | 8 | 0 | 0 |
| Pernambuco | 4724 | 1116 | 2 | | 6953 | | 84 | | 5 | | 5874 | | 78 | | 0 | | 0 | | 4730 | | 1322 | | 40 | | 362 | | 16 | | 2 | | 1187 | 73 | 0 | 0 |
| Piauí | 3426 | 805 | 1 | | 3485 | | 711 | | 8 | | 3396 | | 17 | | 2 | | 0 | | 4006 | | 754 | | 28 | | 358 | | 140 | | 2 | | 965 | 16 | 0 | 0 |
| Rio de Janeiro | 7457 | 1401 | 2 | | 828 | | 3 | | 5 | | 3063 | | 23 | | 0 | | 0 | | 5660 | | 1172 | | 45 | | 121 | | 0 | | 2 | | 1591 | 20 | 0 | 1 |
| Rio Grande do Norte | 3100 | 691 | 2 | | 4551 | | 228 | | 5 | | 4522 | | 1 | | 0 | | 0 | | 3673 | | 741 | | 54 | | 329 | | 36 | | 2 | | 1090 | 1 | 0 | 0 |
| Rio Grande do Sul | 5325 | 1540 | 1 | | 379 | | 2 | | 4 | | 1602 | | 1 | | 0 | | 0 | | 4602 | | 1512 | | 12 | | 118 | | 0 | | 2 | | 955 | 1 | 0 | 0 |
| Rondônia | 2858 | 811 | 1 | | 2294 | | 11 | | 1391 | | 2244 | | 10 | | 2 | | 0 | | 3177 | | 907 | | 41 | | 202 | | 6 | | 25 | | 841 | 11 | 0 | 0 |
| Roraima | 2187 | 614 | 1 | | 2158 | | 275 | | 1278 | | 2342 | | 0 | | 17 | | 0 | | 2288 | | 640 | | 36 | | 326 | | 46 | | 30 | | 1169 | 0 | 2 | 0 |
| São Paulo | 5274 | 1085 | 1 | | 783 | | 43 | | 5 | | 2672 | | 69 | | 0 | | 0 | | 3601 | | 1167 | | 9 | | 137 | | 0 | | 2 | | 762 | 8 | 0 | 0 |
| Santa Catarina | 4090 | 1244 | 1 | | 800 | | 2 | | 6 | | 1496 | | 12 | | 0 | | 0 | | 4467 | | 1074 | | 27 | | 113 | | 7 | | 2 | | 1204 | 70 | 0 | 0 |
| Sergipe | 3222 | 967 | 2 | | 5047 | | 233 | | 5 | | 3441 | | 27 | | 0 | | 0 | | 3479 | | 825 | | 53 | | 297 | | 39 | | 2 | | 1045 | 24 | 0 | 0 |
| Tocantins | 3034 | 815 | 1 | | 2348 | | 1811 | | 55 | | 3128 | | 0 | | 3 | | 0 | | 3419 | | 762 | | 25 | | 243 | | 277 | | 6 | | 722 | 0 | 0 | 0 |
| Brazil | 4594 | 1093 | 1 | | 2465 | | 143 | | 48 | | 3109 | | 41 | | 1 | | 0 | | 4099 | | 1038 | | 33 | | 221 | | 25 | | 3 | | 1103 | 38 | 0 | 0 |

| **YLL (Years of Life Lost)** | | | | | | | | | | | | | | | | | | | | |
| --- | --- | --- | --- | --- | --- | --- | --- | --- | --- | --- | --- | --- | --- | --- | --- | --- | --- | --- | --- | --- |
|  | 1990 | | | | | | | | | | 2017 | | | | | | | | | |
|  | **CVC** | **CHR** | **DEN** | **DIA** | **LEI** | **MAL** | **INR** | **SCH** | **YLF** | **ZIK** | **CVC** | **CHR** | **DEN** | **DIA** | **LEI** | **MAL** | **INR** | **SCH** | **YLF** | **ZIK** |
| Acre | 2458 | 563 | 0 | 3499 | 0 | 809 | 3880 | 0 | 4 | 0 | 2158 | 564 | 12 | 206 | 0 | 13 | 1014 | 0 | 0 | 0 |
| Alagoas | 4001 | 700 | 1 | 10127 | 81 | 1 | 7382 | 26 | 0 | 0 | 4005 | 539 | 16 | 368 | 16 | 1 | 1131 | 12 | 0 | 0 |
| Amapá | 1720 | 259 | 0 | 1016 | 4 | 404 | 1364 | 0 | 3 | 0 | 1920 | 314 | 25 | 121 | 0 | 12 | 824 | 0 | 0 | 0 |
| Amazonas | 1936 | 341 | 0 | 2956 | 5 | 108 | 2307 | 0 | 4 | 0 | 1873 | 330 | 11 | 236 | 1 | 3 | 919 | 0 | 0 | 0 |
| Bahia | 3305 | 537 | 0 | 5476 | 186 | 1 | 4110 | 19 | 1 | 0 | 3502 | 589 | 9 | 252 | 30 | 1 | 993 | 13 | 0 | 1 |
| Ceara | 2979 | 463 | 1 | 6375 | 493 | 1 | 5789 | 2 | 0 | 0 | 3020 | 416 | 18 | 182 | 80 | 1 | 992 | 2 | 0 | 0 |
| Distrito Federal | 2855 | 322 | 0 | 509 | 141 | 7 | 1167 | 19 | 1 | 0 | 2300 | 330 | 10 | 48 | 21 | 1 | 439 | 6 | 0 | 0 |
| Espírito Santo | 4085 | 502 | 0 | 922 | 12 | 2 | 1439 | 16 | 1 | 0 | 4496 | 617 | 22 | 97 | 2 | 1 | 879 | 10 | 0 | 0 |
| Goiás | 3315 | 624 | 0 | 754 | 44 | 27 | 1447 | 31 | 2 | 0 | 3164 | 647 | 29 | 58 | 6 | 1 | 722 | 14 | 0 | 0 |
| Maranhão | 3757 | 498 | 2 | 5678 | 514 | 16 | 6163 | 17 | 11 | 0 | 3338 | 350 | 14 | 285 | 79 | 2 | 1023 | 9 | 0 | 0 |
| Mato Grosso | 2665 | 386 | 0 | 1343 | 129 | 260 | 2021 | 0 | 1 | 0 | 2883 | 541 | 24 | 106 | 20 | 6 | 792 | 0 | 0 | 1 |
| Mato Grosso do Sul | 3672 | 453 | 0 | 892 | 551 | 8 | 1574 | 0 | 1 | 0 | 3890 | 591 | 22 | 138 | 108 | 1 | 914 | 0 | 0 | 0 |
| Minas Gerais | 4529 | 709 | 0 | 811 | 164 | 1 | 1813 | 33 | 1 | 0 | 3632 | 636 | 11 | 73 | 29 | 1 | 937 | 14 | 0 | 0 |
| Pará | 2480 | 410 | 0 | 4020 | 347 | 379 | 3234 | 19 | 3 | 0 | 2609 | 445 | 11 | 177 | 51 | 8 | 1108 | 11 | 0 | 0 |
| Paraíba | 3841 | 594 | 1 | 2443 | 74 | 1 | 3759 | 5 | 0 | 0 | 4134 | 538 | 12 | 151 | 14 | 1 | 1021 | 6 | 0 | 0 |
| Paraná | 4562 | 711 | 0 | 1044 | 2 | 4 | 1587 | 6 | 0 | 0 | 3755 | 792 | 5 | 72 | 0 | 1 | 760 | 4 | 0 | 0 |
| Pernambuco | 4481 | 661 | 1 | 6767 | 82 | 1 | 5746 | 33 | 0 | 0 | 4384 | 888 | 14 | 250 | 15 | 1 | 1070 | 15 | 0 | 0 |
| Piauí | 3223 | 414 | 1 | 3340 | 708 | 3 | 3263 | 14 | 2 | 0 | 3640 | 385 | 8 | 209 | 139 | 1 | 840 | 12 | 0 | 0 |
| Rio de Janeiro | 7120 | 884 | 1 | 747 | 2 | 1 | 2945 | 7 | 0 | 0 | 5189 | 729 | 14 | 63 | 0 | 1 | 1485 | 3 | 0 | 1 |
| Rio Grande do Norte | 2855 | 317 | 1 | 4388 | 227 | 2 | 4396 | 0 | 0 | 0 | 3312 | 360 | 27 | 168 | 36 | 1 | 974 | 0 | 0 | 0 |
| Rio Grande do Sul | 5014 | 981 | 0 | 293 | 2 | 1 | 1483 | 1 | 0 | 0 | 4132 | 993 | 0 | 42 | 0 | 1 | 854 | 0 | 0 | 0 |
| Rondônia | 2703 | 428 | 0 | 2177 | 4 | 904 | 2122 | 1 | 2 | 0 | 2884 | 545 | 20 | 120 | 1 | 13 | 734 | 1 | 0 | 0 |
| Roraima | 2049 | 279 | 0 | 2039 | 267 | 860 | 2220 | 0 | 17 | 0 | 2048 | 307 | 17 | 238 | 33 | 15 | 1052 | 0 | 2 | 0 |
| São Paulo | 4980 | 604 | 0 | 710 | 43 | 1 | 2555 | 19 | 0 | 0 | 3195 | 708 | 1 | 53 | 0 | 1 | 661 | 4 | 0 | 0 |
| Santa Catarina | 3829 | 759 | 0 | 706 | 1 | 2 | 1381 | 8 | 0 | 0 | 4021 | 606 | 6 | 53 | 7 | 1 | 1104 | 8 | 0 | 0 |
| Sergipe | 2996 | 533 | 1 | 4892 | 232 | 1 | 3316 | 15 | 0 | 0 | 3141 | 460 | 21 | 207 | 39 | 1 | 931 | 11 | 0 | 0 |
| Tocantins | 2848 | 411 | 0 | 2208 | 1803 | 50 | 3004 | 0 | 3 | 0 | 3094 | 402 | 11 | 136 | 272 | 4 | 609 | 0 | 0 | 0 |
| Brazil | 4337 | 631 | 1 | 2346 | 141 | 32 | 2987 | 16 | 1 | 0 | 3706 | 614 | 11 | 119 | 24 | 1 | 995 | 7 | 0 | 0 |

| **YLD (Years Lived with Disability)** | | | | | | | | | | | | | | | | | | | | |
| --- | --- | --- | --- | --- | --- | --- | --- | --- | --- | --- | --- | --- | --- | --- | --- | --- | --- | --- | --- | --- |
|  | 1990 | | | | | | | | | | **2017** | | | | | | | | | |
|  | **CVC** | **CHR** | **DEN** | **DIA** | **LEI** | **MAL** | **INR** | **SCH** | **YLF** | **ZIK** | **CVC** | **CHR** | **DEN** | **DIA** | **LEI** | **MAL** | **INR** | **SCH** | **YLF** | **ZIK** |
| Acre | 175.7 | 432.5 | 0.6 | 166.7 | 24.0 | 494.4 | 130.3 | 0.0 | 0.0 | 0.0 | 255 | 359 | 16 | 139 | 23 | 16 | 119 | 0 | 0 | 0 |
| Alagoas | 217.3 | 478.1 | 1.2 | 206.6 | 1.8 | 3.5 | 130.9 | 16.2 | 0.0 | 0.0 | 329 | 363 | 31 | 131 | 0 | 2 | 121 | 21 | 0 | 0 |
| Amapá | 165.0 | 319.5 | 0.9 | 96.4 | 8.1 | 120.2 | 120.4 | 0.0 | 0.0 | 0.0 | 250 | 315 | 22 | 67 | 14 | 13 | 112 | 0 | 0 | 0 |
| Amazonas | 160.8 | 385.5 | 0.7 | 142.9 | 9.9 | 198.1 | 126.3 | 0.0 | 0.0 | 0.0 | 246 | 334 | 17 | 85 | 6 | 11 | 116 | 0 | 0 | 0 |
| Bahia | 217.4 | 442.5 | 0.8 | 169.7 | 4.0 | 3.2 | 129.2 | 8.4 | 0.0 | 0.0 | 354 | 377 | 19 | 160 | 2 | 1 | 117 | 9 | 0 | 0 |
| Ceara | 244.3 | 428.1 | 1.2 | 181.3 | 3.6 | 3.3 | 127.8 | 3.7 | 0.0 | 0.0 | 387 | 372 | 29 | 136 | 1 | 2 | 120 | 5 | 0 | 0 |
| Distrito Federal | 167.0 | 416.5 | 0.8 | 74.4 | 0.8 | 3.5 | 114.8 | 139.4 | 0.0 | 0.0 | 289 | 398 | 18 | 56 | 0 | 2 | 101 | 156 | 0 | 0 |
| Espírito Santo | 243.7 | 441.0 | 1.2 | 95.6 | 1.0 | 4.1 | 118.8 | 7.3 | 0.0 | 0.0 | 493 | 537 | 38 | 108 | 1 | 2 | 136 | 10 | 0 | 0 |
| Goiás | 187.4 | 433.3 | 1.2 | 99.2 | 1.9 | 4.0 | 120.3 | 71.6 | 0.0 | 0.0 | 312 | 408 | 27 | 100 | 1 | 2 | 103 | 72 | 0 | 0 |
| Maranhão | 194.4 | 413.9 | 1.1 | 154.3 | 7.2 | 16.7 | 134.2 | 6.8 | 0.0 | 0.0 | 311 | 347 | 29 | 140 | 13 | 3 | 132 | 9 | 0 | 0 |
| Mato Grosso | 174.8 | 393.5 | 0.9 | 121.7 | 7.4 | 14.9 | 120.7 | 0.0 | 0.0 | 0.0 | 324 | 400 | 23 | 107 | 7 | 2 | 110 | 0 | 0 | 1 |
| Mato Grosso do Sul | 218.4 | 403.7 | 1.0 | 120.6 | 2.6 | 3.7 | 122.9 | 0.0 | 0.0 | 0.0 | 370 | 400 | 24 | 104 | 1 | 2 | 109 | 0 | 0 | 0 |
| Minas Gerais | 262.0 | 457.1 | 0.9 | 100.7 | 1.8 | 3.2 | 118.7 | 43.5 | 0.0 | 0.0 | 431 | 426 | 22 | 115 | 1 | 1 | 104 | 54 | 0 | 0 |
| Pará | 183.9 | 381.2 | 0.9 | 220.3 | 7.1 | 93.5 | 127.0 | 16.4 | 0.0 | 0.0 | 284 | 359 | 24 | 197 | 8 | 6 | 122 | 19 | 0 | 0 |
| Paraíba | 260.3 | 431.9 | 1.0 | 180.8 | 1.2 | 3.1 | 127.0 | 5.8 | 0.0 | 0.0 | 389 | 382 | 26 | 175 | 0 | 2 | 119 | 7 | 0 | 0 |
| Paraná | 251.1 | 484.0 | 0.5 | 110.2 | 1.3 | 3.4 | 119.5 | 3.2 | 0.0 | 0.0 | 402 | 457 | 12 | 99 | 1 | 1 | 102 | 4 | 0 | 0 |
| Pernambuco | 243.2 | 455.0 | 1.0 | 185.1 | 1.8 | 3.3 | 128.1 | 45.3 | 0.0 | 0.0 | 346 | 434 | 26 | 112 | 0 | 1 | 117 | 58 | 0 | 0 |
| Piauí | 203.0 | 390.8 | 0.8 | 144.7 | 3.0 | 4.7 | 132.6 | 3.1 | 0.0 | 0.0 | 366 | 369 | 20 | 148 | 1 | 2 | 125 | 4 | 0 | 0 |
| Rio de Janeiro | 337.1 | 517.1 | 1.3 | 80.2 | 0.4 | 3.1 | 117.9 | 16.4 | 0.0 | 0.0 | 471 | 443 | 31 | 58 | 0 | 1 | 105 | 17 | 0 | 0 |
| Rio Grande do Norte | 245.1 | 373.8 | 1.1 | 163.2 | 0.9 | 3.3 | 125.8 | 0.6 | 0.0 | 0.0 | 360 | 382 | 27 | 161 | 0 | 1 | 115 | 1 | 0 | 0 |
| Rio Grande do Sul | 311.4 | 558.6 | 0.5 | 86.6 | 0.1 | 2.9 | 118.8 | 0.3 | 0.0 | 0.0 | 470 | 519 | 11 | 76 | 0 | 1 | 101 | 0 | 0 | 0 |
| Rondônia | 155.3 | 382.9 | 0.9 | 117.2 | 6.7 | 487.4 | 122.7 | 8.8 | 0.0 | 0.0 | 293 | 362 | 21 | 82 | 5 | 12 | 107 | 10 | 0 | 0 |
| Roraima | 138.1 | 335.4 | 0.8 | 118.7 | 7.6 | 417.9 | 121.4 | 0.0 | 0.0 | 0.0 | 240 | 333 | 19 | 88 | 13 | 15 | 117 | 0 | 0 | 0 |
| São Paulo | 293.9 | 481.8 | 0.9 | 73.6 | 0.5 | 3.1 | 117.3 | 50.2 | 0.0 | 0.0 | 406 | 459 | 8 | 84 | 0 | 1 | 100 | 4 | 0 | 0 |
| Santa Catarina | 261.6 | 485.3 | 0.3 | 94.3 | 0.5 | 3.3 | 115.5 | 3.2 | 0.0 | 0.0 | 446 | 468 | 21 | 60 | 0 | 1 | 100 | 62 | 0 | 0 |
| Sergipe | 225.6 | 433.5 | 1.3 | 155.1 | 0.9 | 3.4 | 125.2 | 11.6 | 0.0 | 0.0 | 339 | 364 | 32 | 90 | 0 | 1 | 114 | 13 | 0 | 0 |
| Tocantins | 185.9 | 404.1 | 0.6 | 139.3 | 8.0 | 5.6 | 124.0 | 0.0 | 0.0 | 0.0 | 324 | 360 | 14 | 107 | 5 | 2 | 114 | 0 | 0 | 0 |
| Brazil | 256.7 | 461.5 | 0.9 | 118.8 | 2.2 | 15.7 | 121.9 | 25.3 | 0.0 | 0.0 | 393 | 423 | 22 | 102 | 2 | 2 | 108 | 31 | 0 | 0 |
